# Cervical Cancer and Pap Smear Awareness and Utilization of Pap Smear Test among Federal Civil Servants in North Central Nigeria

**DOI:** 10.1371/journal.pone.0046583

**Published:** 2012-10-01

**Authors:** Hyacinth I. Hyacinth, Oluwatoyosi A. Adekeye, Joy N. Ibeh, Tolulope Osoba

**Affiliations:** 1 Department of Microbiology Biochemistry and Immunology, Morehouse School of Medicine, Atlanta, Georgia, United States of America; 2 National Center for Primary Care, Morehouse School of Medicine, Atlanta, Georgia, United States of America; 3 Department of Pediatrics, University College Hospital Ibadan, Ibadan, Nigeria; 4 Faculty of Medicine, Department of Public Health, University of Liverpool, Liverpool, England, United Kingdom; IPO, Inst Port Oncology, Portugal

## Abstract

Cervical cancer is a leading cause of cancer death in women in developing countries. A key factor linked to the relatively high levels of cervical cancer in these populations is the lack of awareness and access to preventive methods. This study aimed to determine the level of awareness of cervical cancer and Papanicolaou test (Pap smear test) and factors associated with the utilization of Pap test among female civil servants in Jos. Data was obtained from female workers (n = 388) aged 18–65 years in a Nigerian Federal establishment. Participants were randomly approached and instructed to complete validated questionnaires. Data was analyzed using Chi-square, t-tests and logistic regression analysis to determine if there was an association between variables and identify any predictors of awareness and utilization of the Pap test. Cervical cancer and Pap smear test awareness was 50.9% and 38.6% respectively, with the media as the major source of information. Pap smear test utilization rate was 10.2%, with routine antenatal care (ANC) as the major reason for getting screened. Personal barriers to screening include the lack of awareness, and belief that cervical cancer is not preventable. Opportunistic screening, mass media campaigns and ANC education were suggested as ways of improving awareness and utilization of cervical cancer screening services.

## Introduction

Cervical cancer is a female genital cancer that results from infection with the human papilloma virus, commonly serotypes 16 and 18 [1], [2]. This infection results in transformation of the cervical epithelial cells, first to precancerous lesions and then to frank cancer [3], [4], [5]. Cervical cancer is the fourth leading cause of female genital cancers and sixth cause of female cancer death in the United States [6]. Globally, it is the most common cause of female genital cancer and female cancer deaths [7]. Over 75% of the annual cases of cervical cancer and cervical cancer related morbidity and mortality occur in developing countries [7] usually with less comprehensive cervical cancer prevention programs. In Nigeria, the most recent government estimates put the number of new cases at 25,000 per year [8], while there are no official figures for North Central Nigeria where this study was carried out.

Despite the high mortality from this disease in developing countries, it is preventable and it morbidity and mortality could be greatly reduced using preventative health methods such as safe sexual practice and most importantly the Pap test. The Pap smear test is currently the most widely used approach for preventing cervical cancer, via a 1–3 yearly screening depending on the environment [9], [10]. The benefits of Pap smear test in preventing cervical cancer has been demonstrated by in countries like Finland, Sweden with National screening programs. As a result, these countries are reported to have the lowest incidence and prevalence of cervical cancer and related morbidity and mortality in the world [11], [12]. The high incidence and prevalence of cervical cancer in developing countries such as Nigeria is highly suggestive of health care access issues. For example, a pilot study in Jos, Nigeria reported an estimated annual incidence rate of cervical cancer in 77/1,000 women [13] and mortality of 3000–8000 [14], [15]. These estimates are much higher than those of the United States [6], or Europe [12] where there is regular cervical cancer screening.

Uptake of Pap smear test in Nigeria is very low [16], [17], [18] even when compared with other developing countries [19], [20] further contributing to the reported [8] levels of cervical cancer and its attendant morbidity. It has been reported that almost two thirds of cervical cancer cases in Nigeria present at stage III or later, and that a single one time screen could potentially save more than 6,000 women annually [21]. Various reasons have been suggested for the low utilization of Pap smear test screening in Nigeria. The two main reasons are lack of awareness and poverty [16], [18], [21], [22], [23]. Adewole et al [21] reported a strong relationship between poverty and incidence or prevalence of cervical cancer in low income countries in Africa. Such countries, including Nigeria with high incidence of cervical cancer, were reported to have lower rates of screening for the condition compared with other populations reporting higher levels of awareness. Some investigators concluded that increased awareness may not translate into increased utilization, because studies among healthcare workers, medical students and physicians, who have a high level of awareness demonstrated a lower level of utilization than non-healthcare workers or students [17], [24], [25].

Anecdotal evidence suggests that Federal civil servants in Nigeria are likely to be more educated and posses higher levels of socioeconomic status compared with the general Nigerian population. Thus we hypothesized that the level of awareness of the Pap smear test and cervical cancer is directly related to the level of utilization. Furthermore, that both awareness and utilization would be higher among civil servants than reported for the general population.

## Materials and Methods

The study was approved by the Plateau state Ministry of Health and the University of Liverpool Research Ethics Committee. Due to security concerns, the prisons and police stations were excluded. After estimating the required sample size [17], women ages 18 to 65, who working at a Federal non-healthcare establishment were randomly approached to complete a validated questionnaire [17], [25]. Consenting participants were allowed 1–2 days to complete and return the questionnaire. Of the 450 questionnaires given out, 388 were completed and returned resulting in an 86.2% response rate. The data from the questionnaires were entered into a spreadsheet, categorical data were re-coded into numerical variables and data analysis done using SPSS version 10 for Windows.

Univariate analyses using frequency distribution and means were carried out to describe the characteristics of respondents. Bi-variate analysis was conducted using Chi-square and t-test to investigate associations. Finally, multivariate logistic regression was done to investigate factors that independently predict cervical cancer and Pap smear test awareness and/or utilization of Pap smear test.

## Results

### Socio-demographic characteristics of respondents

A total of 388 questionnaires out of the 450 given out were completed and returned (response rate of 86.2%). The mean age was 38.8±7.3 years (20–61 years), with 65.3% in the 36–55 year old range, 99.0% were Christians and 77.6% are married. Majority (69.7%) of respondents had some form of tertiary education with the mean number of children reported as 4.1±1.6 and ranging from 1–10. More than two-thirds of respondents had 4–6 children, while only 1.0% of reported having more than one sexual partner in the last ten years (table 1), with the mean being 1.01±0.12 partners per respondent.

**Table 1:** Socio-demographic characteristics of respondents and their practice of Pap smear test screening.

Variables	Frequency	Percentage
**Age**		
20–35	120	32.3
36–55	243	65.3
>55	9	2.4
**Marital status**		
Single	55	15.0
Married	284	77.6
Widowed	17	4.6
Divorced	10	2.7
**Educational level**		
Primary	33	9.4
Secondary	73	20.9
Tertiary	244	69.7
**Religion**		
Muslim	4	1.0
Christianity	380	99.0
**Number of children**		
1–3	92	30.6
4–6	193	64.1
7+	16	5.3
**Number of sexual partners**		
Only one	281	98.6
Two or more	4	1.4
**How did you hear about cervical cancer?**		
Media	110	57.6
Hospital	43	22.5
Friends	26	13.6
Relatives	4	2.1
Others	8	4.2
**How did you hear about Pap smear test?**		
Media	69	49.6
Hospital	36	22.5
Friends	15	13.6
Relatives	7	2.1
Others	12	4.2
**When was your last Pap smear test?**		
1–3 years ago	21	80.8
>3 years ago	5	19.2
**How regular is your Pap smear test screening?**		
Regular	26	76.5
Irregular	8	23.5

### Cervical cancer and Pap smear test awareness

Approximately 50.9% of respondents reported having heard about cervical cancer. Twelve and half percent believed the disease to be treatable, while 45.6% felt it could be prevented. Slightly over 38% reported having heard about the Pap smear test and 27.0% said that regular screening with Pap smear test can prevent cervical cancer (Table 2). A little less than 50% of respondents who had heard about Pap smear test got their information from the media, followed by the hospital setting with 25.9% (table 1). Out of the 50.9% (Table 2) of respondents who reported having an awareness of cervical cancer, majority (57.6%) reported obtaining their information from the media, while the second most common source of information was friends and relatives (22.5%). When asked about the risk factors for cervical cancer, 27.6% of respondents identified having multiple sexual partners (MSP) as a risk factor. Early coitarche (20.3%), cigarette smoking (17.2%), having a sexually transmitted disease/infection (14.7%), oral contraceptive use (9.9%), high parity i.e. >3 children (5.7%), and HIV/AIDS (1.7%) were among other factors identified by respondents as risk factors for cervical cancer. Less than four percent had no idea about the risk factors for the disease.

**Table 2:** Showing the level of awareness of cervical cancer, perception of its treatability or preventability, awareness of Pap smear test and its role in preventing cervical cancer among respondents.

Variables	No. of Respondents (%)	
	Yes	No
**Have you heard of cervical cancer?**	191(50.9)	184(49.1)
**Cancer of the cervix can be treated?**	45(12.5)	315(87.5)
**Can cancer of the cervix be prevented?**	160(45.6)	191(54.4)
**Have you ever heard of Pap smear test?**	136(38.6)	216(61.4)
**Can regular screenings with Pap smear test prevent cancer of the cervix?**	89(27.0)	241(73.0)
**Have you ever had a Pap smear test done?**	34(10.2)	300(89.8)

### Utilization of Pap smear test among respondents

Utilization of Pap smear test at least once among this population was 10.2% (table 2), 89.8% reported never having tested. Of the 10.2% that had tested at least once, 65.7% reported testing as part of routine antenatal clinic (ANC) test, 11.4% reported that it was based on a doctor's recommendation, while 22.9% reported that it was their personal initiative. All the respondents who had ever had a Pap smear test did so at a secondary or tertiary health center. 76.5% of respondents, who have ever had a Pap smear test, reported a regular screening pattern i.e. at least once every 1–3 years. About 80% of respondents who have ever had a Pap smear test reported testing within the last 1–3 years, which parallels the result on screening regularity (table 1). Of the 89.8% that had never tested, 14.3% reported inability to locate the facility, 57.1% reported that they had never heard of the test, 6.7% reported being anxious about a positive test result, 4.3% reported that they never thought about having cancer, 9.1% reported that they do not consider it important, 1.9% said they feel the procedure is embarrassing, while 6.7% reported that will rather wait until a later date.

Respondents agreed that the level of awareness can be improved, with 37.0% reporting that this can be done through the provision of information about cervical cancer and Pap smear test during ANC visits. Another 11.0% reported that this can be done using government policies, probably legislations that mandates some form of cervical cancer education in schools, etc. About 37.0% reported that radio and television advertisement can help in improving the level of awareness, while another 15.0% reported that special cancer education program can be used for this purpose.

### Bi-variate analysis

A t-test showed that there was no statistically significant difference in the mean age of respondents who had knowledge of cervical cancer compared with those who did not (39.0±7.8 vs. 38.7±6.3, p = 0.607). The same was true for those who had heard of the Pap smear test compared with those who had not (39.2±7.7 vs. 38.2±7.3, p = 0.267). On the other hand, there was statistically significant difference in the mean age (44.2±7.8 vs. 38.1±7.2 years) of respondents who had a Pap smear test done compared with those who had not, p = 0.02.

Table 3 is a Chi-square test indicating the proportional distribution of respondents and thus the association between awareness about cervical cancer and respondent's age, marital status, educational level, number of children, number of sexual partners, belief that cervical cancer is treatable or preventable, and belief that regular screening with Pap smear test can prevent cancer. It indicates that cervical cancer awareness was associated with marital status (with singles having the highest level of awareness), educational status (with respondents who possess tertiary education having the highest level of awareness) and perception about cervical cancer,(with individuals who believe that cervical cancer is treatable or preventable by having regular Pap smear test, having the highest level of awareness), p<0.01.

**Table 3:** Indicates the proportional distribution of respondents that have or have not heard about cervical cancer by certain socio-demographic variables and perception of cervical cancer.

Variables	Response *n*(%)		Chi-Square Value	*P*-value
	Yes	No		
**Age category (years)**			0.13	0.93
20–35	58(50)	58(50)		
36–55	114(49)	120(51)		
>55	4(44)	5(56)		
**Marital status**			16.41	<0.01
Single	29(57)	22(43)		
Married	149(52)	135(48)		
Widowed	4(24)	113(76)		
Divorced	0(0)	10(100)		
**Educational level**			46.36	<0.01
Primary	0(0)	33(100)		
Secondary	34(47)	39(53)		
Tertiary	149(62)	91(38)		
**Number of children**			2.11	0.34
1–3	53(58)	39(42)		
4–6	91(48)	97(52)		
≥7	8(50)	8(50)		
**Number of sexual partners**			5.34	0.02
Only one sexual partner	162(58)	119(42)		
Two or more sexual partners	0(0)	4(100)		
**Believe that cervical cancer is treatable**			28.02	<0.01
Yes	41(91)	4(9)		
No	150(49)	156(51)		
**Awareness of cervical cancer preventability**			81.45	<0.01
Yes	128(81)	30(219)		
No	59(32)	128(68)		
**Regular Pap smear test can prevent cervical cancer**			41.75	<0.01
Yes	73(82)	16(18)		
No	97(42)	135(58)		

Similarly, table 4 shows that there is a relationship between awareness about Pap smear test and respondent's age (with women older than 55 years tend to be more aware about the Pap smear test). Also, Proportionally more married women, women with tertiary education, those with 4–6 children, those who believe it is a preventable disease, and those who believe that regular Pap smear test is the way to prevent cervical cancer, had heard about Pap smear test. In addition, table 5 shows that respondent's perception of the disease was associated with utilization of Pap smear test. Those respondents who believed that cervical cancer is not treatable, but preventable via regular screening were more likely to have had a Pap smear test. Similarly, there was a relationship between utilization of Pap smear test and respondent's educational level (respondents with tertiary education were more likely to have had a Pap smear test) and their awareness about Pap smear test (p<0.01). Other factors significantly associated with utilization of Pap smear test includes; age (with older women (>55 years) more likely to have had a Pap smear test, p<0.01), awareness about Pap smear test (p<0.01) and general perception of Pap smear test as a preventative procedure for cervical cancer.

**Table 4:** Association between respondent's level of awareness about Pap smear test there socio-demographic characteristic and perception of cervical cancer.

Variables	Response *n*(%)		Chi-Square Value	*P*-value
	Yes	No		
**Age category (years)**			5.42	0.06
20–35	31(28)	80(72)		
36–55	84(39)	132(61)		
>55	5(56)	4(44)		
**Marital status**			18.71	<0.01
Single	13(26)	37(74)		
Married	109(42)	152(58)		
Widowed	0(0)	13(100)		
Divorced	0(0)	10(100)		
**Educational level**			10.60	0.01
Primary	4(16)	21(84)		
Secondary	21(30)	48(70)		
Tertiary	102(44)	127(56)		
**Number of children**			18.67	<0.01
1–3	29(33)	59(67)		
4–6	89(49)	91(51)		
≥7	0(0)	16(100)		
**Number of sexual partners**			2.76	0.09
Only one sexual partner	110(41)	158(59)		
Two or more sexual partners	0(0)	4(100)		
**Awareness of cervical cancer preventability**			67.74	<0.01
Yes	95(61)	61(39)		
No	32(17)	151(83)		
**Regular Pap smear test can prevent cervical cancer**			71.65	<0.01
Yes	68(80)	17(20)		
No	63(27)	169(73)		

**Table 5:** Shows the proportional difference among respondents based on utilization and practice of Pap smear test.

Variables	Response *n*(%)		Chi-Square Value	*P*-value
	Yes	No		
**Age category (years)**			29.02	<0.01
20–35	0(0)	100(100)		
36–55	22(12)	188(88)		
>55	4(50)	4(50)		
**Marital status**			1.73	0.63
Single	4(8)	43(92)		
Married	21(9)	230(91)		
Widowed	0(0)	9(100)		
Divorced	0(0)	10(100)		
**Educational level**			12.19	0.01
Primary	0(0)	29(100)		
Secondary	0(0)	65(100)		
Tertiary	25(12)	185(88)		
**Number of children**			2.54	0.28
1–3	8(10)	72(90)		
4–6	22(13)	151(87)		
≥7	0(0)	16(100)		
**Number of sexual partners**			-	-
Only one sexual partner	25(10)	253(90)		
Two or more sexual partners	0(0)	0(0)		
**Believe that cervical cancer is treatable**			5.35	0.02
Yes	0(0)	45(100)		
No	29(11)	240(89)		
**Awareness of cervical cancer preventability**			18.05	<0.01
Yes	24(17)	121(83)		
No	5(3)	170(97)		
**Regular Pap smear test can prevent cervical cancer**			8.67	0.01
Yes	16(19)	69(81)		
No	18(7)	223(93)		
**Ever heard about the Pap smear test**			52.39	<0.01
Yes	34(25)	101(75)		
No	0(0)	186(100)		

### Multi-variate statistical analysis

Multi-variate analysis was done using multiple logistic regression models to investigate the predictors of awareness of cervical cancer, Pap smear test, and utilization of Pap smear test in the study population. The result of the analysis showed that none of the variables (age category, marital status, educational level, number of children, source of cervical cancer information, belief in the treatability of cervical can or preventability of cervical cancer using regular Pap smear test) significantly predict level of awareness of cervical cancer. A similar analysis was done with the dependent variable being awareness about Pap smear test and it showed that the only significant predictor of awareness was a belief that cervical cancer is a preventable disease (p = 0.04, CI = 0.02–0.98, p = 0.04). On the other hand, a similar analysis with utilization of Pap smear test as the dependent variable did not reveal any significant predictor.

## Discussion

This study, done among female federal civil servants showed that cervical cancer awareness was 50.9%, (table 2) a value that is slightly less than what was reported among women in South Eastern Nigeria [18]. And significantly lower than was reported (71% awareness) among female undergraduates in South Western Nigeria [17]. The significant difference with the study among female undergraduates might be because this study involved a large portion of the “lay public” as opposed to study by Ayinde et al which was done among female undergraduates, a good portion of whom were medical student. As seen in table 2, 38.6% of respondents were aware of Pap smear test, which is a much higher level of awareness when compared with what was reported university undergraduates [17] or sexually active women non-civil service women [22]. But it is noted that this level of awareness was lower than had been reported among Kuwaiti, Jordanian, Indian and Saudi Arabian (developing countries like Nigeria) female outpatient population [19], [20], [26], [27]. About half of the respondents still cited the media followed by hospitals as their source of information about cervical cancer or Pap smear test. Thus the media plays an important role in disseminating health educational information with the next most common source being the hospitals. Other authors have shown that the role of the media in disseminating health maintenance information cannot and should not be discounted when health education is being carried out [28], [29]. A health education program about cervical cancer that incorporates the media could be very impactful in our environment. The National Health Service (NHS) stated that the involvement of prominent public figures like movie stars or music artists can go a long way. For instance, they reported an increase in uptake of cervical cancer screening when a prominent movie star was brought in to tell her story about how cervical cancer has impacted her life negatively [30]. Thus a well funded media campaign could change the current picture in Nigeria. Since this study did not attempt to make a distinction between the type of media vehicle, inference cannot be made as to which will be more effective. But anecdotal evidence suggests that most public offices in Nigeria have a radio and as such it could be inferred that campaigns done via radio might have a wider reach. About 40% of respondents in this study were aware of the risk factor for cervical cancer. This is slightly higher than was reported among sexually active women from South Eastern Nigeria [22]. Knowledge of whether cancer of the cervix is preventable was higher among this study population than reported among commercial sex workers in a part of Nigeria [23]. The percentage of respondents who believed that cervical cancer was preventable seems to be closely related to the level of awareness about cervical cancer and Pap smear test (Table 2). The number of children that respondents had as at the time of this study yielded a mixed picture when measured with the level of awareness about cervical cancer (not statistically significant) and Pap smear test (statistically significant). One conclusion could be that, since Pap smear test is done routinely as part of ANC care, it is possible that the knowledge of Pap smear test was gotten from there. Although it is not clear why none of the women with ≥7 children were aware of Pap smear test as seen in table 3, if the prior explanation were to be the case. The relationship between level of awareness about Pap smear test and cervical cancer was statistically significant. A similar result was documented among lay women in Nigeria [22], possibly because information about both is usually provided at the same time. The investigators expected respondents with multiple sexual partners to be more aware about cervical cancer and Pap smear test, but this was not the case in our study, which support earlier report from a study among commercial sex workers in Nigeria [23].

### Utilization of Pap smear test

The level of utilization of Pap test smear test among respondents in this study was 10.2% (table 2), slightly higher than was reported among female state civil servants from a similar population [31], and much higher than the 4.4% that was reported in Northern Nigeria among female healthcare workers [16], and female undergraduates [17]. The level of utilization of Pap smear test in this study population was also higher than was reported among health care workers in Jos [32], although lower than was reported among female healthcare workers from South Eastern Nigeria [32]. Older to middle aged women were more likely to have had a Pap smear test (table 4). Lack of awareness about Pap smear test was the most commonly cited reason for not having had a Pap smear test. This result is similar to that obtained among Hispanic American females and inner city girls, who also cited lack of awareness as the their major reason for not having had a Pap smear test [33], [34]. A similar factor also surfaces in studies done among Middle Eastern [19], [20], [26] and Nigerian women [17], [18], [22]. Contrary to a prior study [24], table 4 shows that in this study, there was a significant association between awareness about the test and its utilization (p<0.01). Similarly, there was also a significant relationship between respondent's level of education and utilization of Pap smear test, with utilization being higher among women with tertiary education compared with those who have secondary or primary education, which is in contrast to a previous report [22].

Majority of those who had done the test reported doing it as part of their antenatal care. This is an important finding because many women go through the antenatal clinic. A report indicates that a onetime screening will result in the identification of 8000 new cases of invasive cancer per annum [21] and ANC visit provides a perfect opportunity for such onetime screening. Only 11.9% of respondents mentioned that a doctor or nurse's recommendation was the reason they had a test. This number is not encouraging because it could mean that healthcare practitioners are not recommending the test, at least not as much as needed to make a difference. This is in support of findings among Hispanic women and other minority female populations in the United States, where one of the reasons given for not having done the Pap smear test was because the doctor/healthcare provider did not recommend it [33], [34].

A bright side from this study is the fact that about 76% of those who reported that they have done a Pap smear test said that they do so regularly (which is every 1–3 years), which means that if the importance of initiating screening is properly communicated and healthcare workers can get the women to start screening, they are likely to keep it up. Another good news from this study is the fact that about 81% of respondent said that their last Pap smear test was within the last three years. This implies that the majority of respondents who engage in Pap smear test tend to abide by the recommended protocol.

Finally, the regression model showed a statistically significant relationship between awareness about Pap smear test and belief or knowledge that cervical cancer is preventable. Thus it is possible that women who have come to understanding that cervical cancer is a preventable disease are more likely to actively seek information about the means of doing so. This also has health education implications because it means that it is not just enough to provide information about cervical cancer, but to also let women know that it is preventable and tell them how, where and when to obtain such preventable services.

## Conclusions

This study shows a lower level of awareness of cervical cancer (50.9%), and Pap smear test (38.6%) compared with reports from other parts of Nigeria. The major source of information about cervical cancer and Pap smear test was the media. There was no statistically significant relationship between educational level and level of awareness. Although awareness about cancer of the cervix was higher among single women, there was no statistically significant relationship between marital status and Pap smear test awareness, perception or utilization.

Educational level, awareness about Pap smear test, treatability of cervical cancer and preventability of cervical cancer, are among factors that showed a statistically significant relationship with utilization of Pap smear test. The level of awareness and utilization from this study was higher than seen among female state civil servants. This may have something to do with a higher socioeconomic status (not tested in this study) among Federal civil servants who tend earn more in terms of salary than state civil servants. Finally, it is the opinion of the authors that there is need for intense, well focused cervical cancer education in order to increase awareness because we have seen from this study that improved awareness can translate into improved utilization.

### Recommendations

Based on our findings, we recommend that an intense public health campaign be conducted on a recurring basis; providing cervical cancer education with emphasis on its etiology, risk factors and methods of prevention. Public service announcements promoting Pap smear test utilization and its benefits should be carried out by the government and other non-governmental organizations and schools alone or in conjunction with the government. Policies that encourage doctors to recommend the procedure to their female patients who meet the necessary requirements for commencing Pap smear test screening should be instituted. Policies should also be put in place that encourage or as appropriate mandate that all women coming for antenatal services are provided with the options of a Pap smear test. Cervical cancer education needs to be properly packaged so that the message delivered is complete. Finally facilities capable of carrying out Pap smear test be established, and more opportunistic screenings be done so that as many eligible women as possible can get at least one Pap smear test.

### Limitations

This study represents an improvement on the results reported from an earlier study. But there are some limitations that could affect the overall conclusion that has been drawn from the results. A good portion of the study sample had a higher literacy level (tertiary education) than the general population and this might have biased the outcome of this study. Some information about cervical cancer and Pap smear test was provided in the information section of the questionnaire, there is no way to ascertain if the reported level of awareness was prior to their getting the questionnaire or not, although attempt was made to word the questions to refer to prior knowledge. This also could have favorably biased the result of the study. This study did not measure socioeconomic status, however it may have been a reasonable variable to explore as it might have been the reason for the lower levels of awareness and/or utilization obtained in other populations compared with this one. There is no way of validating the accuracy of the information provided by the respondents. Thus larger population studies need to be conducted.
